# Long-term health consequences and costs of changes in alcohol consumption in England during the COVID-19 pandemic

**DOI:** 10.1371/journal.pone.0314870

**Published:** 2025-01-16

**Authors:** Joshua Card-Gowers, Sadie Boniface, Jamie Brown, Loren Kock, Alexander Martin, Lise Retat, Laura Webber

**Affiliations:** 1 HealthLumen Ltd, London, United Kingdom; 2 Centre for Behaviour Change, Research Department of Clinical, Educational and Health Psychology, University College London, London, United Kingdom; 3 Institute of Alcohol Studies, London, United Kingdom; 4 Tobacco and Alcohol Research Group, Department of Behavioural Science and Health, University College London, London, United Kingdom; 5 AstraZeneca, Barcelona, Spain; Oswaldo Cruz Foundation: Fundacao Oswaldo Cruz, BRAZIL

## Abstract

**Background and aims:**

The COVID-19 pandemic led to changes in alcohol consumption in England. Evidence suggests that one-fifth to one-third of adults increased their alcohol consumption, while a similar proportion reported consuming less. Heavier drinkers increased their consumption the most and there was a 20% increase in alcohol-specific deaths in England in 2020 compared with 2019, a trend continuing through 2021 and 2022. This study aimed to quantify future health, healthcare, and economic impacts of changes in alcohol consumption observed during the COVID-19 pandemic.

**Methods:**

This study used a validated microsimulation model of alcohol consumption and health outcomes. Inputted data were obtained from the Alcohol Toolkit Study, and demographic, health and cost data from published literature and publicly available datasets. Three scenarios were modelled: short, medium, and long-term, where 2020 drinking patterns continue until the end of 2022, 2024, and 2035, respectively. Disease incidence, mortality, and healthcare costs were modelled for nine alcohol-related health conditions. The model was run from 2020 to 2035 for the population of England and different occupational social grade groups.

**Results:**

In all scenarios, the microsimulation projected significant increases in incident cases of disease, premature mortality, and healthcare costs, compared with the continuation of pre-COVID-19 trends. If COVID-19 drinking patterns continue to 2035, we projected 147,892 excess cases of diseases, 9,914 additional premature deaths, and £1.2 billion in excess healthcare costs in England. The projections show that the more disadvantaged (C2DE) occupational social grade groups will experience 36% more excess premature mortality than the least disadvantaged social group (ABC1) under the long-term scenario.

**Conclusions:**

Alcohol harm is projected to worsen as an indirect result of the COVID-19 pandemic and inequalities are projected to widen. Early real-world data corroborate the findings of the modelling study. Increased rates of alcohol harm and healthcare costs are not inevitable but evidence-based policies and interventions are required to reverse the impacts of the pandemic on alcohol consumption in England.

## Introduction

The coronavirus pandemic (COVID-19) has had multiple direct and indirect effects on population health. In England, one of the indirect effects of the pandemic has been changes in alcohol consumption. Drinking patterns shifted partly due to strict enforcement of several non-pharmaceutical interventions that included restrictions on movement and social contact, stay-at-home orders, and the closure of services and non-essential businesses, collectively known as ‘lockdowns.’

Initial evidence from the UK suggested that approximately a fifth to a third of people reported drinking more alcohol during lockdown than previously, with a similar proportion drinking less [1]. Alcohol duty receipts to 2021 indicated a small increase in the total volume of alcohol cleared for sale or receipts from alcohol duty during the acute phases of the pandemic [2]. Because some people drank more while others drank less during the pandemic, individual self-reported consumption is valuable to understand population subgroups which are obscured in aggregated data on alcohol sales.

The Alcohol Toolkit Study (ATS) collects data monthly and has found that the proportion of people drinking at increasing or higher risk levels (as measured by the Alcohol Use Disorders Identification Test) increased with the onset of the pandemic and has remained above pre-pandemic levels into 2024 [3, 4]. In line with these results, Sohi et al. 2022’s systematic review highlighted that the number of people drinking at harmful levels increased in the United Kingdom during the lockdowns in 2020 and 2021 [5].

Importantly, these rises in the prevalence of increasing and higher-risk drinking were not spread evenly across society [6]. The rise in increasing and higher-risk drinking was particularly apparent in disadvantaged groups [3, 7] and heavier drinkers [7, 8]. This has clear health consequences, especially given that COVID-19 itself highlighted and exacerbated existing health inequalities [9, 10].

Changes in alcohol consumption during the pandemic have been followed by increases in alcohol harm in England. Between 2019 and 2021, there was a 27.5% increase in alcohol-specific deaths in England [11] and provisional data indicate this has persisted into 2022 (data currently available up to May 2022) [12]. There was also a 13.5% increase in unplanned hospital admissions for alcohol-related liver disease in 2020 in England [7], which persisted throughout 2021 (data currently available to December 2021) [7, 12]. There is additional evidence that substance use treatment outcomes have worsened during the pandemic [13]. These changes were triggered by an acute crisis and followed a sharp reduction in healthcare utilisation and access to healthcare [14–16], however, the healthcare system has not recovered. By the end of 2021, emergency department attendances were close to pre-pandemic levels, but outpatient referrals, waiting lists, and ambulance call response times are still severely impacted and national targets are not met [17–20].

Research on the medium-to-longer term impacts of the trends of alcohol consumption is necessary to indicate the future disease burden of alcohol-related conditions, and to inform policy responses. There are over 200 health conditions linked to alcohol, and many of these are chronic conditions including cardiovascular diseases and seven types of cancer [21]. This means that the health consequences of changes in alcohol consumption may take several years to be fully realised. Microsimulation has the utility to understand future trends in risk factors and diseases and to inform future policies and outcomes. This study aimed to quantify the projected longer-term impact of changes in alcohol consumption during the COVID-19 pandemic on epidemiological (incidence of certain alcohol-related diseases, premature mortality) and economic outcomes (healthcare costs), as well the impact on inequalities in England, using a peer-reviewed and validated microsimulation model [22–25].

## Materials and methods

### Overview of the microsimulation model

This study adapted a previously published microsimulation model [22–27] to project the health impacts of the changes in alcohol consumption observed during 2020 and 2021 as an indirect effect of the COVID-19 pandemic [5] by incorporating new data inputs and developing different COVID-19 pandemic-related scenarios, detailed below. Microsimulation is an advanced method for modelling non-communicable diseases (NCDs) due to its capacity to simulate entire populations at an individual level over time.

100 million individuals were cycled through the microsimulation annually between 2020 and 2035, and outputs of interest were presented from 2022 to 2035. The end year was chosen to allow sufficient time to see the long-term impacts of increased alcohol consumption on the development of the modelled diseases. As the microsimulation progressed by year, changes in the population developed at an individual level (for example individual and population ageing, births, and deaths, and changing risk factors for the incidence of the diseases of interest). Nine alcohol-associated diseases were explored. These alcohol-related diseases included six cancers, (breast, colorectal, liver, mouth, oesophageal, and throat cancer), hypertension, liver cirrhosis, and stroke. These were selected as some of the most prevalent alcohol-related diseases with strong evidence to support independent relative risks of alcohol. Outputs included changes in cumulative incidence of the diseases of interest, associated mortality, and direct healthcare costs.

The model was run in three hypothetical future scenarios and one baseline scenario. Outputs from each of the test scenarios were compared with the baseline scenario to develop an understanding of the impact of changes in alcohol consumption through and beyond the COVID-19 pandemic. The test scenarios assumed that the increases in alcohol consumption during the COVID-19 pandemic were maintained over short- medium- and long-term beyond 2022. The baseline ‘no-change’ scenario assumed that levels of alcohol consumption stayed at 2019 levels throughout the simulation.

#### Data inputs and assumptions

**Fig 1:**
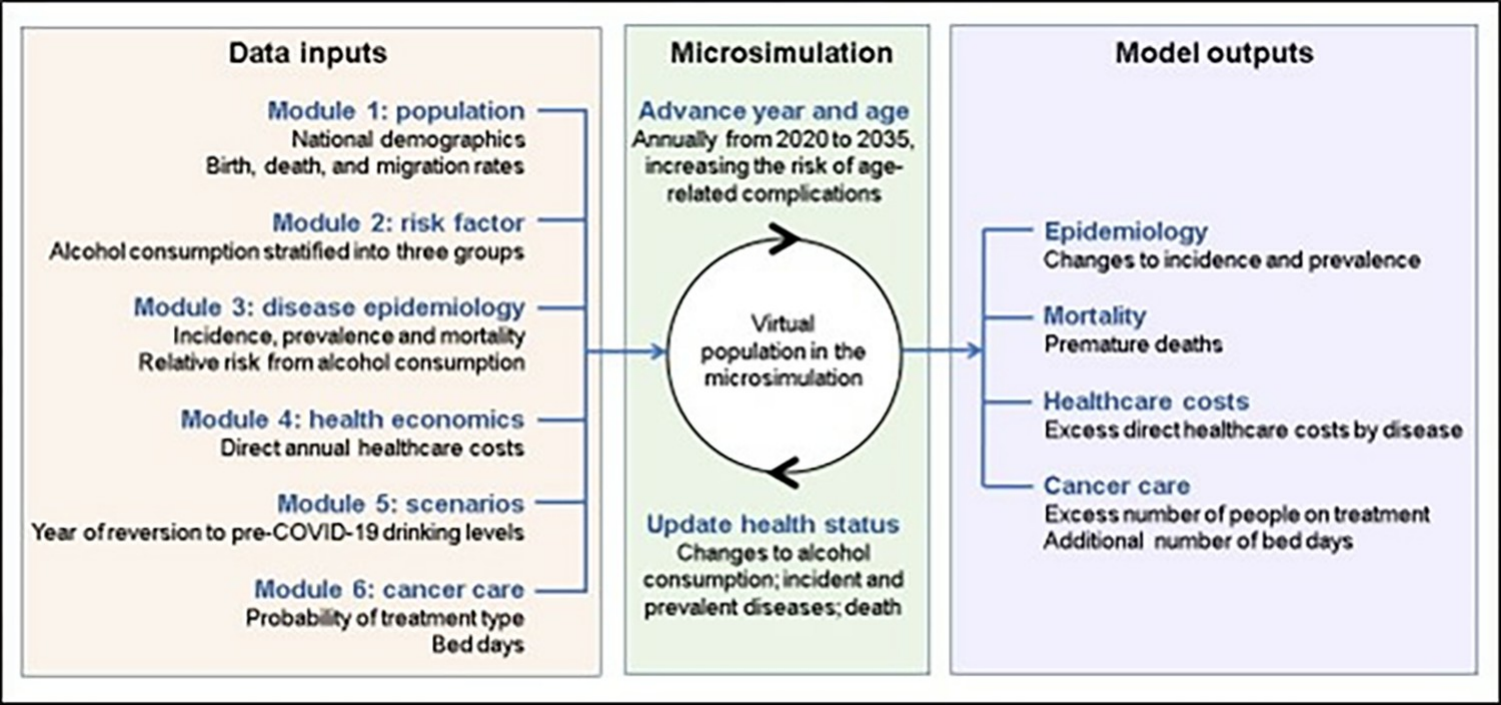
Illustration of the microsimulation model.

The inputs of the microsimulation six modules (Fig 1): population, risk factors, disease epidemiology, economics, scenarios, and cancer care. Data sources used in each of these six modules are shown in the Supplementary material.

*Module 1*: *Population*. The microsimulation consists of a virtual population aged 0–110 that is representative of the population of England. At baseline (2020), each individual in the model was assigned an age and sex in line with England’s national statistics [28]. Sex and age were assigned based on the number of male and females in each single-year age group in the ONS and the virtual population matched population statistics in 2021. The microsimulation allowed for changes in population through ageing, births and deaths throughout the study period, and population projections accounted for migration [28]. Individuals could die of any cause (through background death rate) or from a specific disease.

The model assigned socioeconomic status (SES) grades to individuals according to the Approximated Social Grades (ASGs), ABC1 (less disadvantaged SES grades) and C2DE (more disadvantaged SES grades), based on occupation (sourced from the UK National Census, 2011) [29] as described by the National Readership Survey [30] (NRS, S1 Table). The social grade ABC1 includes individuals who likely hold professional or managerial positions and have completed higher education, and the C2DE group includes those who are manual workers, casual workers, and the unemployed. As the 2011 census did not capture SES data below age 16 and above age 65, it was assumed that 50% of individuals below the age of 16 belong to the ABC1 and C2DE SES categories and that the distribution of SES in the age 60–64 population was representative of the population aged between 65 and 110 years due to the lack of social grouping data after retirement age. SES was assigned probabilistically. It was assumed that individuals in the model would stay within the assigned SES group for the duration of the study period.

The size of the virtual UK population of the microsimulation was chosen to be 100 million to accurately project the incidence of the rarer cancers included in the model. All results were then scaled to the size of the projected population in England.

*Module 2*: *Risk factors*. Alcohol consumption (units per week) was assigned to each individual based on data extracted from the ATS. One unit of alcohol is equivalent to 10ml of pure alcohol. Individuals were categorised into three levels of risk, in line with alcohol risk categories used by the UK Health Security Agency (formerly Public Health England) [31] to provide projections of policy-related alcohol groupings. These categories include distributions of individuals, such that each individual has their own discrete alcohol consumption value:

**High-risk alcohol consumption** (>50 units/week in males, >35 in females)

**Increasing-risk alcohol consumption** (14–50 units/week in males, 14–35 units/week in females)

**Low-risk alcohol consumption** (<14 units/week in males and females)

The inputs for alcohol consumption were collected for discrete groups, being males and females in three age groups (15–39, 40–59, and 60+) and two SES groups (ABC1, and C2DE). Each of these groups has a different alcohol consumption distribution which was used to assign discrete alcohol consumption values to individuals in the model, depending on their age, sex and SES (Supplementary material). This grouping was chosen to maintain a sufficient sample size whilst allowing a reasonable level of granularity. Individuals can change alcohol consumption group as they cross an age boundary (40 and 60) in the microsimulation, which occurs following the distributions of alcohol consumption trends in the individuals’ specific sex and SES group. Population-level alcohol consumption was held static at 2019 levels in the baseline scenario, and alcohol was the only modelled modifiable risk factor. The proportion of people in the low, increasing, and high-risk alcohol consumption groups within each age, sex, and SES group remained the same through the modelled time period. Assigned alcohol intake was associated with a relative risk of developing the alcohol-related diseases of interest in each year of the microsimulation.

*Module 3*: *Disease epidemiology*. Nine alcohol-related diseases were modelled: hypertension, liver cirrhosis, stroke and five cancers (colorectal, breast, oesophageal, liver, mouth, and throat). These diseases were chosen as some of the most common NCDs linked to alcohol consumption, with a significant burden on healthcare systems. For the modelled cancers, the data on incidence, mortality, and survival were extracted by age and sex from the Cancer Research UK (CRUK) online data and statistics portal [32]. The relative risks of alcohol consumption on the incidence of modelled cancers were extracted from a meta-analysis conducted by Bagnardi et al [33]. For hypertension, liver cirrhosis, and stroke, extensive literature reviews were conducted to source incidence, prevalence, mortality, survival, and relative risk data by sex and age group (S4–S6 Tables). An individual’s relative risk of contracting one of the modelled diseases is based on their alcohol consumption in any given year, and socioeconomic status was not one of the variables for complication relative risk.

*Module 4*: *Health economics*. Annual direct costs for each modelled disease were calculated by multiplying the annual direct cost per patient, which were identified through literature searches, by the number of prevalent cases output in the model in a particular year (S7–S15 Tables). Costs were not discounted through the simulation period and are shown in 2021 GBP.

*Module 5*: *Scenarios*. The health and cost impacts of three ‘test’ scenarios were compared with a baseline ‘no change’ scenario.

**Baseline ‘no change’ scenario’,** in which alcohol consumption within specific age, sex and SES groups was assumed to have remained stable at pre-pandemic levels throughout the whole of the simulation (2020–2035).

**‘Short-term’ scenario,** in which the alcohol consumption patterns of 2020 and 2021 remained throughout 2022, before returning to pre-pandemic levels at the start of 2023.

**‘Medium-term’ scenario,** in which the alcohol consumption patterns of 2020 and 2021 remain until the end of 2024, before returning to pre-pandemic levels at the beginning of 2025.

**‘Long-term’ scenario,** in which the alcohol consumption patterns of 2020 and 2021 remain indefinitely (from 2022 until the end of the microsimulation in 2035).

Inputs for the three scenarios as well as the ‘no change’ scenario were derived from data from the ATS (Supplementary material), a national representative monthly cross-sectional survey of alcohol use behaviour in England [34]. The probabilities of an individual moving from one alcohol consumption group to another in the short, medium, and long-term scenarios were also calculated using pre- and post-Covid-19 alcohol consumption levels in the ATS (S2, S3 Tables).

*Module 6*: *Cancer care*. The probability of an individual receiving chemotherapy, radiotherapy, or a tumourectomy, was extracted from CRUK [32] and the National Cancer Registration and Analysis Service (NCRAS) [35].

#### Data outputs and analysis

The model ran from 2020 to 2035; outputs were presented from 2022 onwards (the year in which the analysis took place). Outputs of the model included the incidence of the nine diseases of interest, the associated direct healthcare costs, resource utilisation, associated premature mortality (deaths under 75) and the number of additional individuals needing cancer therapies (tumourectomy, radiotherapy, or chemotherapy) (Fig 1).

These outputs were presented as cumulative values by year and were calculated in the total population of England, as well as in one of the subpopulations (higher and lower SES groups), under the three hypothetical future scenarios and the baseline scenario.

The impact of each scenario was calculated through comparison with outputs from the no-change baseline analysis.

## Results

### Impact of changes in alcohol consumption on the population-wide incidence and mortality of nine alcohol-related diseases

**Fig 2:**
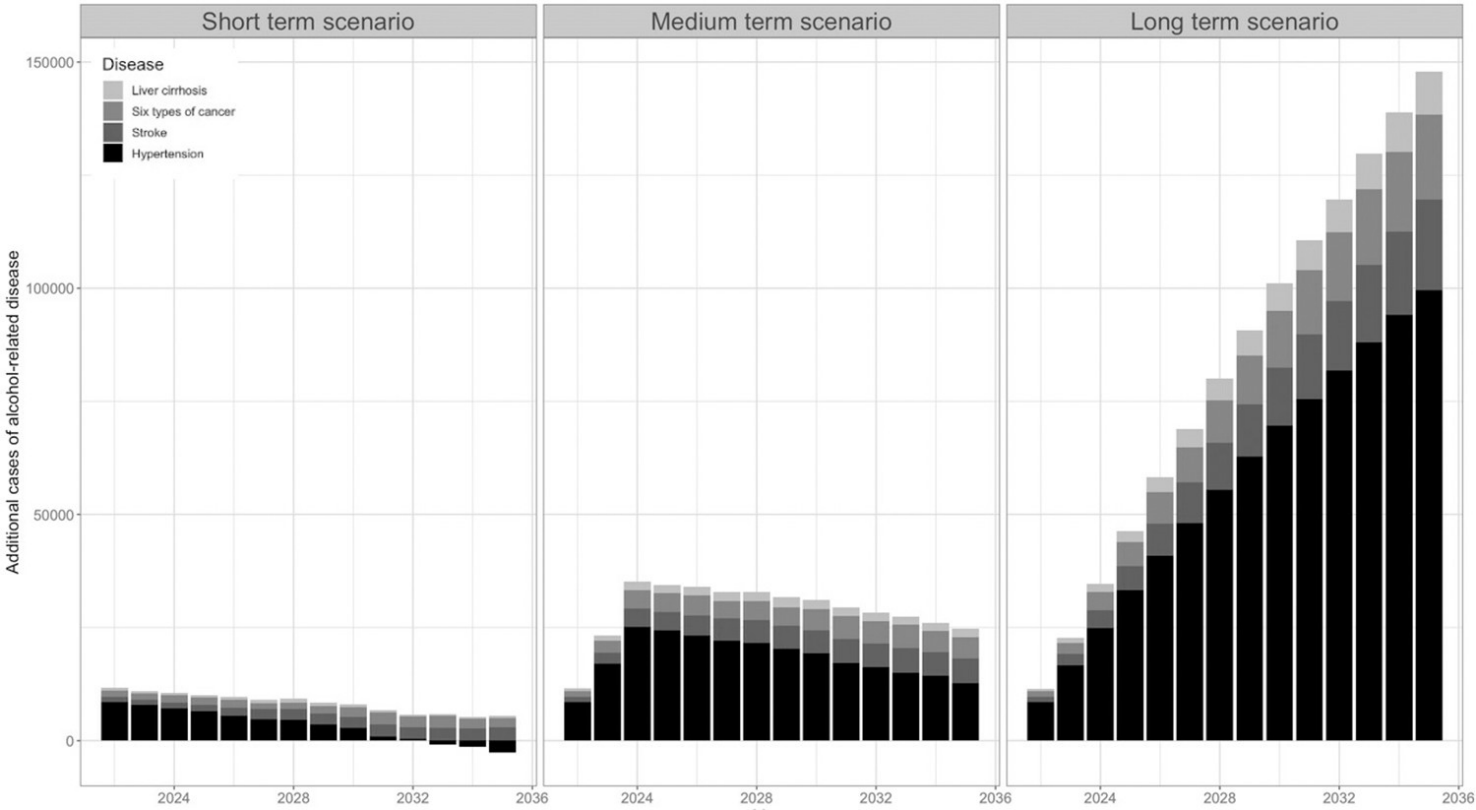
Impact of changing patterns of alcohol-consumption observed during the Covid-19 pandemic on cumulative incidence of alcohol-related disease if maintained over short-, medium- or long-term, in England between 2022 and 2035. ‘Short-term’ scenario assumes the alcohol consumption patterns of 2020 and 2021 remained throughout 2022, before returning to pre-pandemic levels at the start of 2023. ‘Medium-term’ scenario, assumes the alcohol consumption patterns of 2020 and 2021 remain until the end of 2024, before returning to pre-pandemic levels at the beginning of 2025. ‘Long-term’ scenario, in which the alcohol consumption patterns of 2020 and 2021 remain indefinitely (from 2022 until the end of the microsimulation in 2035).

**Fig 3:**
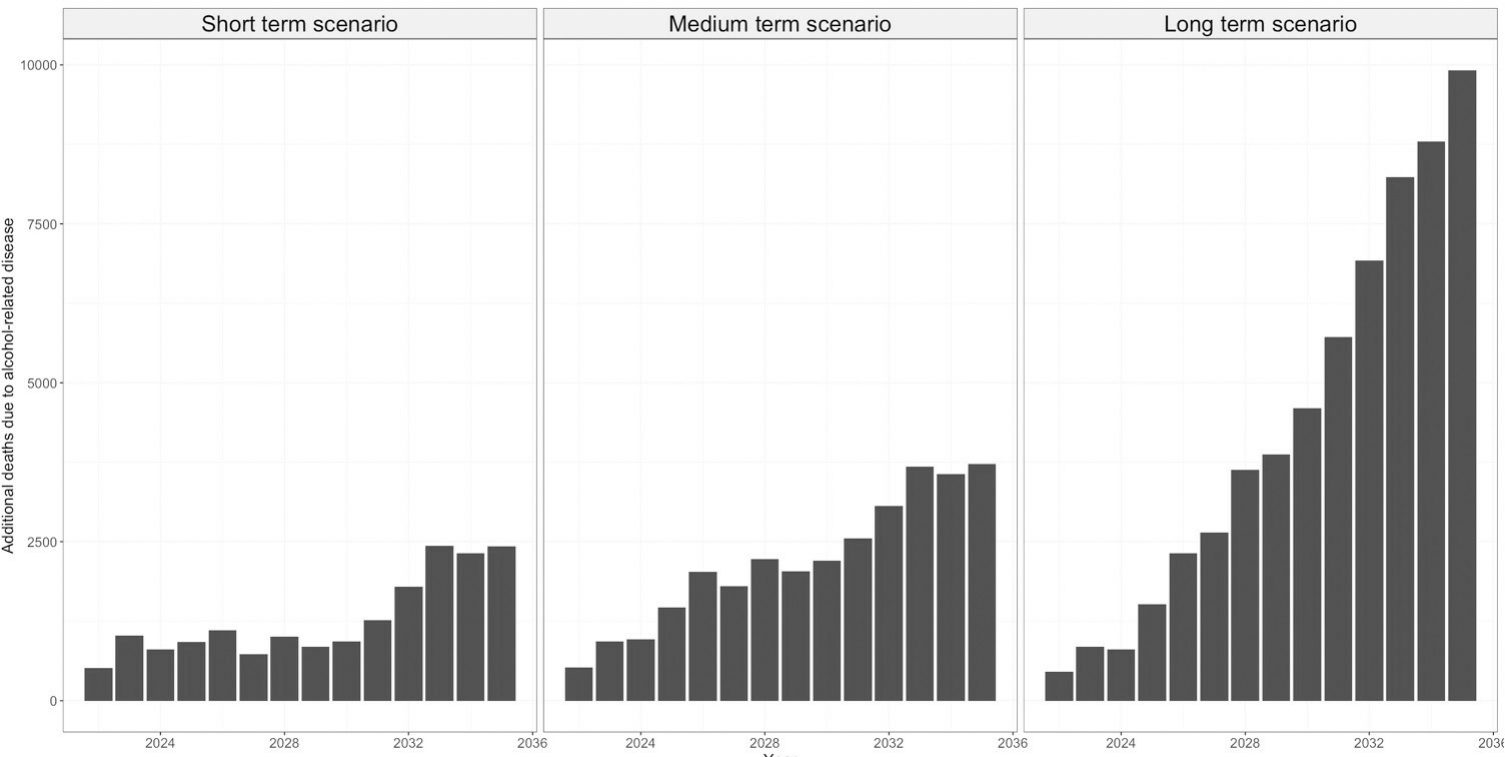
Impact of changing patterns of alcohol-consumption observed during the Covid-19 pandemic on cumulative associated mortality if maintained over short-, medium- or long-term, in England between 2022 and 2035. ‘Short-term’, ‘medium-term’ and ‘long-term’ scenarios are defined as above.

In all three scenarios, the model projected an increase in the incident cases of disease and mortality compared with the no-change baseline scenario (Figs 2 and 3).

#### Cumulative incidence of nine alcohol-related diseases

The microsimulation projected that between 2022 and 2035, there would be a cumulative increase in the incidence of the nine alcohol-related diseases of 2,860, 24,706 and 147,892 in the short- medium- and long-term scenarios respectively, compared to the no change baseline. There would be a cumulative excess incidence of 10, 87, and 507 disease cases per 100,000 between 2022 and 2035 in the short- medium- and long-term scenarios respectively.

For both the short-term and medium-term scenarios, the incidence of the nine alcohol-related diseases studied reverted towards baseline incidence over time, after alcohol consumption had returned to pre-pandemic levels (at the start of 2023 and the start of 2025, respectively). In the short-term scenario, in which alcohol consumption returned to pre-pandemic levels in 2023, the incidence of hypertension was predicted to fall below that of the no-change scenario by 2035, most likely due to premature deaths occurring in those with conditions associated with excess alcohol consumption.

In all three scenarios, the alcohol-related disease of greatest incidence was hypertension, followed by stroke. Combined, the six types of alcohol-related cancers included in the model accounted for 12.7% of the overall increased incidence in modelled alcohol-related diseases.

#### Premature mortality from nine alcohol-related diseases

The microsimulation predicted that between 2022 and 2035, there would be a cumulative increase in premature mortality associated with the nine alcohol-related diseases of 2,431, 3,725 and 9,914 in the short- medium- and long-term scenarios respectively, compared to the no change baseline scenario (Fig 3).

### Impact on inequalities

Individuals belonging to the lower occupational grade group (C2DE) were projected to face worse outcomes than individuals belonging to the higher occupational grade group (ABC1) per 100,000 people if pandemic alcohol consumption patterns continue. Excess cumulative incidence of the nine diseases was projected to be 242 and 265 per 100,000 in the ABC1 and C2DE populations, respectively, by 2035 under the long-term scenario compared to the no-change scenario (Table 1). This equates to the lower occupational social grade group having a 9.5% higher cumulative incident disease cases per 100,000 population than the higher occupational grade group. Furthermore, there were 36% more additional cumulative premature deaths in the C2DE population than in the ABC1 population per 100,000 under the long-term scenario.

**Table 1:** Excess cumulative incidence of diseases projected in the ABC1 and C2DE population, per 100,000, by the year of reversion to pre-COVID-19 drinking patterns and by 2035.

Population	ABC1					C2DE				
Scenario	Short-term		Medium-term		Long-term	Short-term		Medium-term		Long-term
Year	2022	2035	2024	2035	2035	2022	2035	2024	2035	2035
Breast cancer	1 (±1)	3 (±3)	2 (±1)	5 (±3)	10 (±3)	1 (±1)	0 (±3)	2 (±1)	1 (±3)	8 (±3)
Colorectal cancer	0 (±1)	2 (±3)	1 (±1)	3 (±3)	7 (±3)	0 (±1)	-1 (±3)	1 (±1)	0 (±3)	5 (±3)
Hypertension	13 (±2)	-2 (±9)	42 (±4)	24 (±9)	164 (±9)	13 (±4)	-6 (±9)	46 (±4)	22 (±9)	176 (±9)
Liver cancer	0 (±0)	0 (±1)	0 (±0)	0 (±1)	2 (±1)	0 (±0)	0 (±1)	1 (±0)	1 (±1)	2 (±1)
Liver cirrhosis	1 (±0)	1 (±2)	3 (±1)	3 (±2)	15 (±2)	1 (±1)	1 (±2)	3 (±1)	3 (±2)	18 (±2)
Mouth cancer	0 (±0)	0 (±1)	1 (±0)	1 (±1)	5 (±1)	1 (±0)	1 (±1)	2 (±0)	2 (±1)	7 (±1)
Oesophageal cancer	1 (±0)	0 (±1)	2 (±1)	1 (±1)	6 (±1)	0 (±1)	1 (±1)	2 (±1)	2 (±1)	8 (±1)
Stroke	2 (±1)	3 (±5)	5 (±2)	7 (±5)	31 (±5)	4 (±2)	7 (±5)	9 (±2)	12 (±5)	38 (±5)
Throat cancer	0 (±0)	0 (±1)	1 (±0)	0 (±1)	2 (±1)	0 (±0)	0 (±1)	1 (±0)	0 (±1)	3 (±1)

Note: figures in brackets in this table refer to the standard error in excess cumulative incidence of diseases projected per 100,000 individuals within ABC1 and C2DE populations, by year.

**Fig 4:**
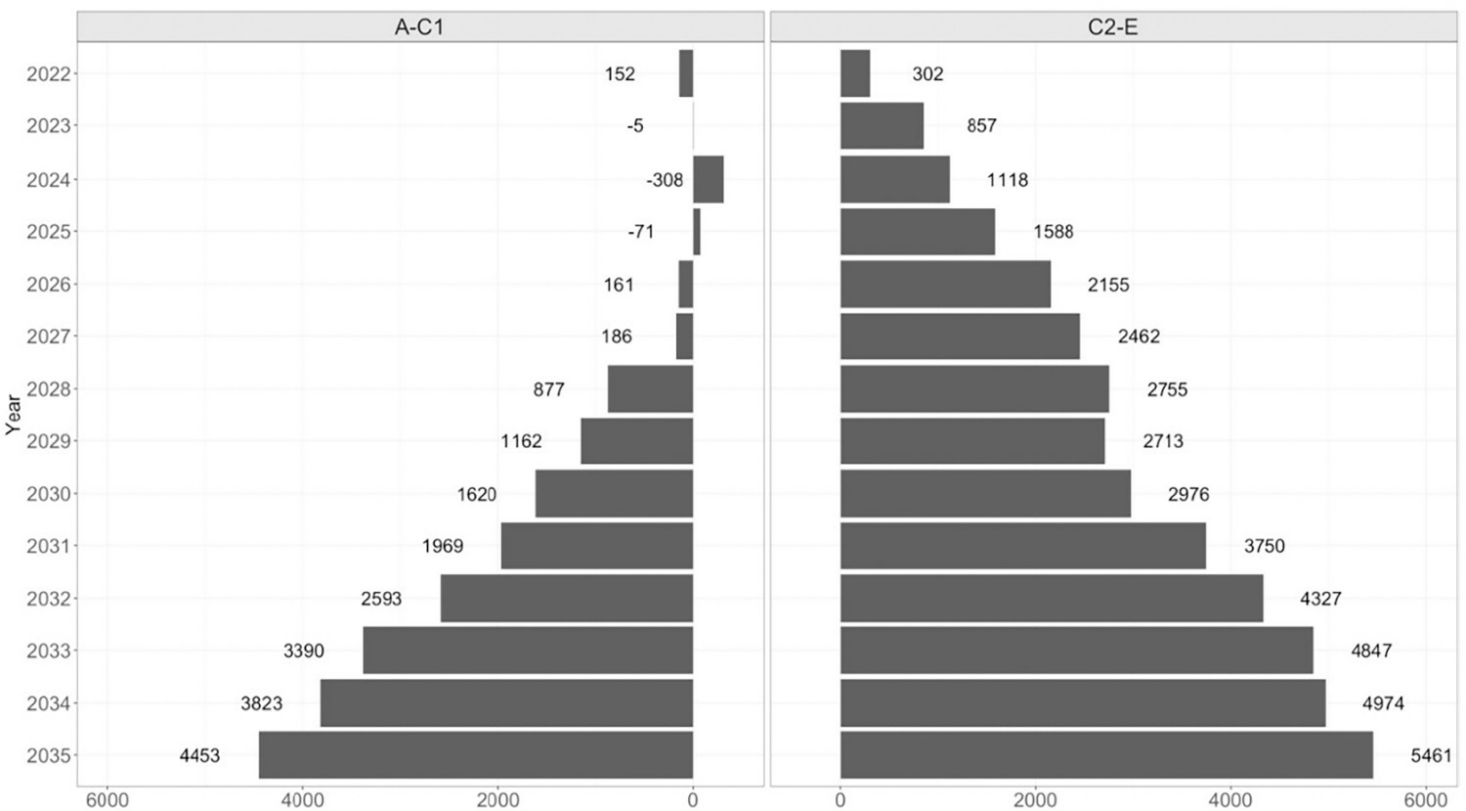
Number of excess premature deaths in the ABC1 and C2DE populations, between 2022 and 2035, under the long-term scenario compared to the baseline scenario. The ‘long-term’ scenario models a scenario in which the post-COVID-19 alcohol consumption patterns of 2020 and 2021 remain indefinitely (from 2022 until the end of the microsimulation in 2035).

By the end of the microsimulation, cumulative excess premature deaths in C2DE population were predicted to be 36% greater than in the in the ABC1 population, under the long-term scenario (Fig 4).

### Healthcare costs

**Fig 5:**
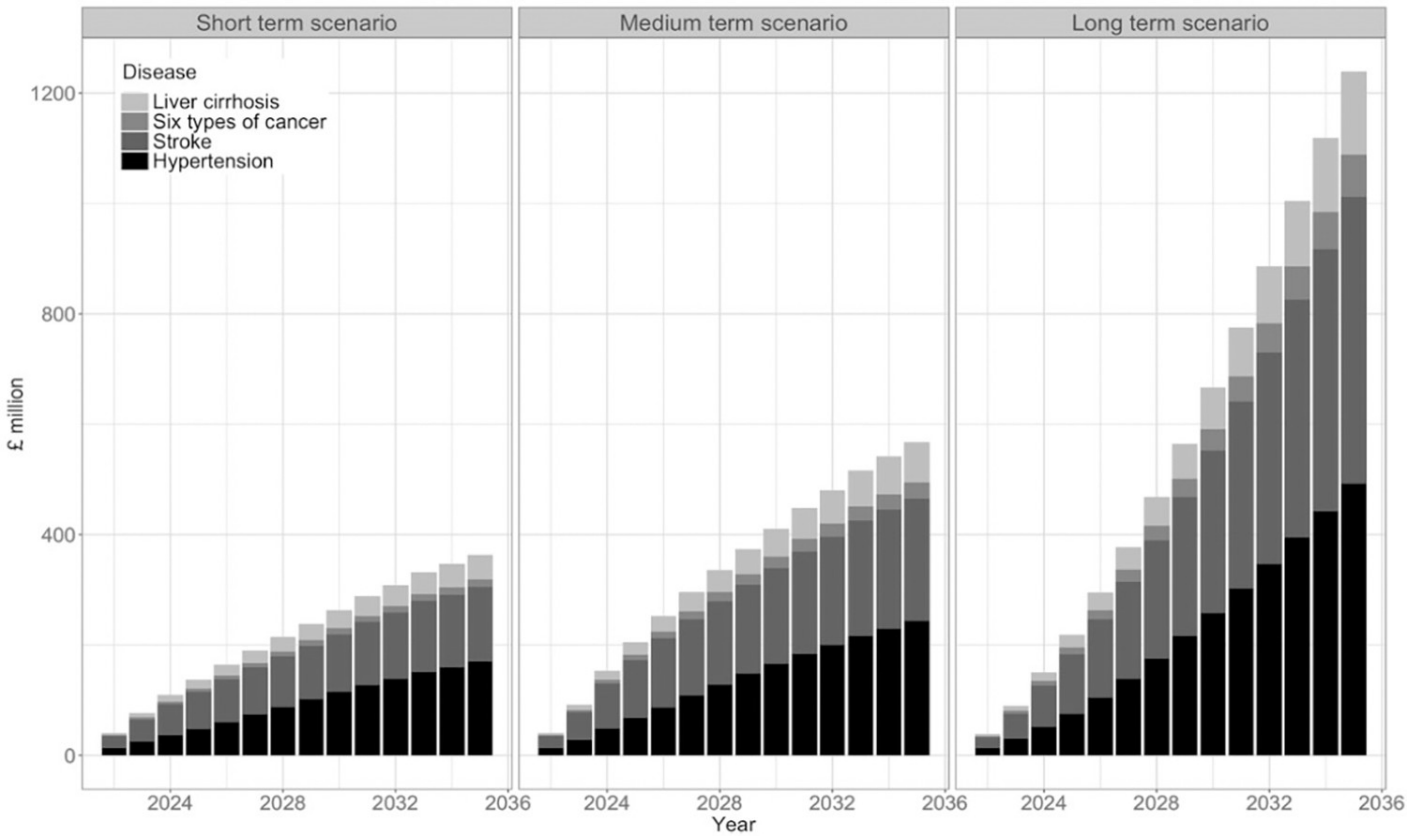
Impact of changing patterns of alcohol-consumption observed during the COVID-19 pandemic, on cumulative direct costs of treating alcohol-related disease, in England, between 2022 and 2035. All results are presented in 2021 GBP (£). Additional costs are reflective of the additional prevalence of disease in any given year.

Total direct healthcare costs for the selected diseases modelled by 2035 were £369 million in the short-term scenario, £568 million in the medium-term scenario, and £1.2 billion in the long-term scenario (Fig 5). Although alcohol-related cancer accounted for around 10% of the additional incidence of disease cases, it was projected to incur a disproportionately large healthcare cost.

## Discussion

In all three scenarios, alcohol harm in England is set to worsen substantially as an indirect result of the pandemic. Depending on how long the changes in alcohol consumption persist, this is between 2,860 and 147,892 additional cases of nine alcohol-related diseases by 2035, which is likely to impact the health service through greater utilisation resulting from poorer health (S1 Fig). This is projected to lead to between 2,431 and 9,914 extra premature deaths, impacting those less well-off in society the most. Finally, the extra costs to the National Health Service are estimated to be between £363 million and £1.2 billion. All the health outcomes modelled to 2035 are chronic non-communicable diseases which develop over many years, meaning the increases in alcohol harm are not inevitable and can be prevented.

The results of this study are consistent with a further modelling study for England using the Sheffield Alcohol Policy Model. The Sheffield model includes a greater range of alcohol-related diseases and explored a different range of possible future consumption scenarios, but their main scenario projected that over the next 20 years, there will be 207,597 additional alcohol-attributable hospital admissions, 7,153 additional alcohol-attributable deaths, and additional costs to the NHS of £1.1 billion [36]. The findings of this study are also consistent with real-world increases already observed in hospital admission rates for alcoholic liver disease in England, rising from 43.3 per 100,000 individuals in 2019/20 to 49.4 per 100,000 in 2022/3, and in mortality rate from alcoholic liver disease in individuals aged under 75 in England, rising from 9.1 per 100,000 individuals in 2019 to 11.6 per 100,000 in 2022 [37]. Between 2019 and 2021, there was a 27.4% increase in alcohol-specific deaths in the UK (9,641 deaths in 2021, 8,974 deaths in 2020, and 7,565 deaths in 2019) [11]. Although the UK and Ireland were the only countries in Europe not to see a fall in alcohol use with the immediate onset of the pandemic, and with many countries outside of Europe also reporting overall decreases in alcohol consumption during the pandemic including South Africa, Columbia and Mexico, real-world data from the USA, Japan, and Australia and modelling studies in the USA suggest that the UK is not in a unique position facing increases in harm from alcohol as an indirect effect of the COVID-19 pandemic [38–43].

### Strengths and limitations

Strengths of this research include the use of a well-validated microsimulation modelling approach which enabled the projection of increased alcohol consumption in millions of individuals over time. This allows for differences in disease incidence, prevalence, mortality, and costs to be identified between different groups. Flexibility in the microsimulation model was leveraged to account for some of the uncertainty surrounding the expected duration of changes in drinking patterns that occurred during the pandemic by modelling scenarios that consider three possible future durations of changes in alcohol consumption.

This study investigated nine alcohol-related health conditions. Considering there are over 200 health conditions linked to alcohol as well as wider economic and societal harms (including injuries to self and others and traffic collisions), the full extent of the future health and economic burden of alcohol consumption is not presented. Additionally, the impacts of alcohol on other aspects of health and wellbeing including mental health, quality of life, and harms to others such as the effects on children and families, drink-driving collisions, or domestic violence were not included in this study. Improvements in the surveillance and monitoring of these conditions and events would make it possible to include a wider range of outcomes in future modelling studies.

The primary source of alcohol consumption data was the ATS. As with many surveys, in response to the pandemic, the survey mode changed from face-to-face to telephone delivery from April 2020 onwards, which may have impacted the measurement of alcohol consumption. However, earlier research using the ATS investigated the comparability of the telephone and face-to-face survey modes in a sensitivity analysis and concluded it is reasonable to compare data from before and during lockdowns [3]. Alcohol consumption in the microsimulation was assumed to be static over time and has not attempted to model any underlying mechanisms to explain the causation of increased alcohol consumption or the interaction between different agents which may effect alcohol consumption trends in the future. Additionally, alcohol was the only modelled risk factor, and other risks such as obesity and smoking were not included in the analysis. Studies have demonstrated that smoking has a bi-directional relationship with alcohol, and increased alcohol intake has been demonstrated to lead to obesity, therefore, the impacts of elevated alcohol consumption on the modelled diseases may be greater than projected in this study [44, 45]. Furthermore, there is a likelihood that the outcomes of the long-term scenarios could be worse than projected if higher-risk drinking behaviours were re-normalised.

### Implications

The Alcohol Health Alliance UK is calling on the UK Government to launch an independent review of alcohol harm [46] and the results of this study underline the urgency of such a review. Extensive evidence already exists on the health benefits and cost-effectiveness of various alcohol control policies, for example: investing in alcohol treatment, pricing interventions (taxation and minimum unit pricing), controlling availability, and regulating alcohol marketing [6, 47]. In England, a number of alcohol-related policies have been updated or introduced in recent years, including alcohol duty reviews, and the inclusion of public health campaigns such as “Dry January” and “Drink Free Days” under the UK Government’s official guidelines for alcohol consumption [48, 49]. There have also been calls for minimum unit pricing, already introduced in Scotland and Wales, to be further extended to England [50]. As well as having the strongest evidence base, such policies can complement other ongoing UK Government policy agendas and would contribute to the health, social and economic recovery from the pandemic. These interventions and policies will also disproportionately benefit more deprived groups in England, who currently experience the highest rates of alcohol harm despite similar levels of consumption [51]. Additionally, there may also be opportunities for traditional and electronic screening and brief interventions, with a persistent rise in the proportion of people trying to restrict their alcohol consumption [4], and a rise in the use of apps to reduce alcohol use during acute phases of the pandemic [52].

### Future research

Future research should focus on predicting the impact of a range of complementary public policy interventions to reduce harm from alcohol. This could examine the health and economic impacts of interventions on socio-economic and regional inequalities, to inform policymakers about the potential ways to narrow these inequalities, contributing to the UK Government’s ‘levelling up’ agenda. The longer-term indirect impacts of the COVID-19 pandemic on alcohol harm now overlap with difficult economic circumstances in England (the ‘cost-of-living crisis’, characterised by high levels of inflation and an anticipated economic recession), which were not addressed in this study. Future research could model the impact of the cost-of-living crisis on alcohol use and harm, and further modelling could produce a broader range of outputs, such as years of life lost and carer burden. The most recent alcohol consumption data could also be integrated into the scenarios to update the projections. More research is also needed to identify the relative risks of alcohol on health conditions, and the impact of heavy episodic drinking on alcohol-related disease. This will enable a more sophisticated modelling approach that has particular relevance in the context of understanding health inequalities related to alcohol consumption [53].

## Conclusions

Changes in alcohol consumption are one indirect effect of the COVID-19 pandemic in England and are projected to result in a significantly increased health and economic burden in England from alcohol-related diseases. If drinking patterns do not revert to pre-COVID-19 patterns, the disease burden will be far higher. The increases in alcohol harm projected are not inevitable and can be prevented if evidence-based policies and interventions are implemented as part of a coherent strategy to reduce harm from alcohol, prevent inequalities from widening further, and reduce the pressure on the healthcare system.

## Supporting information

S1 Fig Percentage increase in alcohol-related diseases by socio-economic status group, compared with no change baseline, 2022 to 2035. (DOCX)

S1 Table The percentage of men and women of each age group who are in the A-C1 and C2-E socioeconomic status categories in the 2011 census. (DOCX)

S2 Table The probabilities of an individual belonging to a certain alcohol consumption group, by age, sex, and SES, at the start of the baseline model (using data from ATS). (DOCX)

S3 Table The probabilities of transition to a higher alcohol consumption group, by age, sex, and SES, at the start of the COVID-19 scenarios. (DOCX)

S4 Table Sources for epidemiology data. (DOCX)

S5 Table Survival rates from disease data sources. (DOCX)

S6 Table Relative risks in microsimulation data sources. (DOCX)

S7 Table Cost of colorectal cancer data sources. (DOCX)

S8 Table Cost of breast cancer data sources. (DOCX)

S9 Table Cost of oesophageal cancer data sources. (DOCX)

S10 Table Cost of liver cancer data sources. (DOCX)

S11 Table Cost of mouth cancer data sources. (DOCX)

S12 Table Cost of liver cirrhosis data sources. (DOCX)

S13 Table Cost of throat cancer data sources. (DOCX)

S14 Table Cost of stroke data sources. (DOCX)

S15 Table Cost of hypertension data sources. (DOCX)
