# Supplementary material for: Long-term health consequences and costs of changes in alcohol consumption in England during the COVID-19 pandemic
Source: PLoS One. 2025 Jan 16;20(1):e0314870. doi: 10.1371/journal.pone.0314870 (PMC11737736; doi:10.1371/journal.pone.0314870)

*S1 Fig. Percentage increase in alcohol-related diseases by socio-economic status group, compared with no change baseline, 2022 to 2035.*


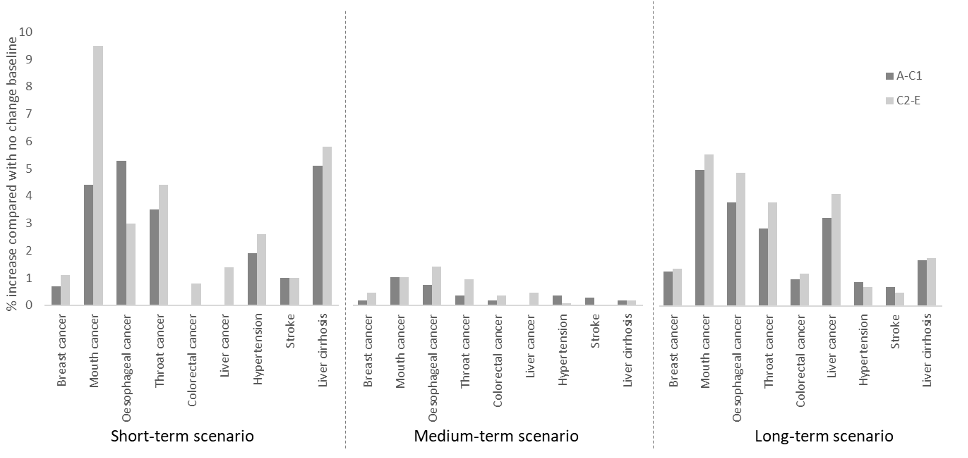

Supplement: S1 Fig — (DOCX) [file pone.0314870.s001.docx]
