# Supplementary material for: Long-term health consequences and costs of changes in alcohol consumption in England during the COVID-19 pandemic
Source: PLoS One. 2025 Jan 16;20(1):e0314870. doi: 10.1371/journal.pone.0314870 (PMC11737736; doi:10.1371/journal.pone.0314870)
Supplement: S1 Table — (DOCX) [file pone.0314870.s002.docx]

*S1 Table.: The percentage of men and women of each age group who are in the A-C1 and C2-E socioeconomic status categories in the 2011 Census*

| Age | Male | | Female | |
| --- | --- | --- | --- | --- |
|  | **A-C1** | **C2-E** | **A-C1** | **C2-E** |
| 16 to 19 | 48.91% | 51.09% | 48.83% | 51.17% |
| 20 to 21 | 52.94% | 47.06% | 52.25% | 47.75% |
| 22 to 24 | 51.58% | 48.42% | 50.35% | 49.65% |
| 25 to 29 | 53.63% | 46.37% | 54.53% | 45.47% |
| 30 to 34 | 56.56% | 43.44% | 58.49% | 41.51% |
| 35 to 39 | 56.15% | 43.85% | 57.86% | 42.14% |
| 40 to 44 | 53.66% | 46.34% | 55.93% | 44.07% |
| 45 to 49 | 52.00% | 48.00% | 55.28% | 44.72% |
| 50 to 54 | 50.61% | 49.39% | 54.01% | 45.99% |
| 55 to 59 | 49.60% | 50.40% | 53.10% | 46.90% |
| 60+ | 48.17% | 51.83% | 50.50% | 49.50% |
