# Supplementary material for: Long-term health consequences and costs of changes in alcohol consumption in England during the COVID-19 pandemic
Source: PLoS One. 2025 Jan 16;20(1):e0314870. doi: 10.1371/journal.pone.0314870 (PMC11737736; doi:10.1371/journal.pone.0314870)
Supplement: S2 Table — (DOCX) [file pone.0314870.s003.docx]

S2 Table. The probabilities of an individual belonging to a certain alcohol consumption group, by age, sex, and SES, at the start of the baseline model (using data from ATS)

| Age | Alcohol consumption group | A-C1 | | C2-E | |
| --- | --- | --- | --- | --- | --- |
|  |  | Male | Female | Male | Female |
| 15-39 | low | 89.5% | 95.8% | 93.4% | 97.6% |
|  | medium | 9.8% | 3.2% | 5.6% | 1.9% |
|  | high | 0.6% | 1.0% | 1.0% | 0.5% |
| 40-59 | low | 81.6% | 92.3% | 84.4% | 96.1% |
|  | medium | 17.1% | 6.6% | 13.8% | 2.8% |
|  | high | 1.3% | 1.2% | 1.8% | 1.1% |
| 60+ | low | 82.0% | 93.7% | 89.8% | 97.8% |
|  | medium | 16.7% | 5.6% | 9.1% | 1.5% |
|  | high | 1.3% | 0.8% | 1.1% | 0.6% |

Data calculated from ATS data^[[1]](#footnote-1)^

1. The risk consumption categories used in this study were defined by weekly alcohol unit consumption as:

   Male: Low risk: <=14, medium risk: >14 and <50, high risk: >=50

   Female: Low risk: <=14, medium risk: >14 and <35, high risk: >=35 [↑](#footnote-ref-1)
