# Supplementary material for: Long-term health consequences and costs of changes in alcohol consumption in England during the COVID-19 pandemic
Source: PLoS One. 2025 Jan 16;20(1):e0314870. doi: 10.1371/journal.pone.0314870 (PMC11737736; doi:10.1371/journal.pone.0314870)
Supplement: S3 Table — (DOCX) [file pone.0314870.s004.docx]

S3 Table. The probabilities of transition to a higher alcohol consumption group, by age, sex, and SES, at the start of the COVID-19 scenarios

| Age | Transition state | A-C1 | | C2-E | |
| --- | --- | --- | --- | --- | --- |
|  |  | **Male** | **Female** | **Male** | **Female** |
| 15-39 | Low to medium | 4.845% | 2.662% | 4.644% | 2.160% |
|  | Medium to high | 7.044% | 28.946% | 16.510% | 50.371% |
| 40-59 | Low to medium | 6.330% | 4.457% | 3.917% | 5.118% |
|  | Medium to high | 5.481% | 17.859% | 6.135% | 44.021% |
| 60+i | Low to medium | 3.812% | 0.989% | 5.998% | 2.108% |
|  | Medium to high | 8.748% | 8.992% | 11.096% | 8.600% |

Data calculated from ATS data^[[1]](#footnote-1)^

1. The risk consumption categories used in this study were defined by weekly alcohol unit consumption as:

   Male: Low risk: <=14, medium risk: >14 and <50, high risk: >=50

   Female: Low risk: <=14, medium risk: >14 and <35, high risk: >=35 [↑](#footnote-ref-1)
