# Supplementary material for: Long-term health consequences and costs of changes in alcohol consumption in England during the COVID-19 pandemic
Source: PLoS One. 2025 Jan 16;20(1):e0314870. doi: 10.1371/journal.pone.0314870 (PMC11737736; doi:10.1371/journal.pone.0314870)
Supplement: S4 Table — (DOCX) [file pone.0314870.s005.docx]

S4 Table. Sources for epidemiology data

| Sources for data used in simulations | | | |
| --- | --- | --- | --- |
| Disease | | **Reference** | **ICD codes** |
| Colorectal Cancer | Incidence | CRUK, 2021: Bowel Cancer: 2016-2018 [1] | C18-C20 |
|  | Prevalence* |  |  |
|  | Mortality |  |  |
| Breast Cancer | Incidence | CRUK, 2021: Breast Cancer: 2016-2018 [2] | C50 |
|  | Prevalence* |  |  |
|  | Mortality |  |  |
| Oesophageal Cancer | Incidence | CRUK, 2021: Oesophageal Cancer: 2016-2018 [3] | C15 |
|  | Prevalence* |  |  |
|  | Mortality |  |  |
| Liver Cancer | Incidence | CRUK, 2021: Liver Cancer: 2016-2018 [4] | C22 |
|  | Prevalence* |  |  |
|  | Mortality |  |  |
| Mouth Cancer | Incidence | CRUK, 2021: Head and Neck Cancer: 2016-2018 [5] | C00-C07, C09 |
|  | Prevalence* |  |  |
|  | Mortality |  |  |
| Throat Cancer | Incidence | CRUK, 2021: Head and Neck Cancer: 2016-2018 [5] | C10-C13, C32 |
|  | Prevalence* |  |  |
|  | Mortality |  |  |
| Liver Cirrhosis | Incidence | Ratib et al. 2014 [6] | K70.3, K71.7, K72.1, K74.4-74.6, K76.6 |
|  | Prevalence |  |  |
|  | Mortality | European mortality database [7] |  |
| Stroke | Incidence | BHF Compendium, 2020, table 2.15 [8] | I60-I69 |
|  | Prevalence | Health Survey for England, 2017, table 1 [9] |  |
|  | Mortality | BHF Compendium, 2021, table 1.1 [10] |  |
| Hypertension | Incidence | Calculated | I10-I15 |
|  | Prevalence | BHF Compendium, 2021, table 5.6 [10] |  |
|  | Mortality | Non-terminal |  |

*Calculated from incidence, mortality and survival

## References

1. Cancer Research UK. *Bowel Cancer Statistics*. [Internet] [cited 2022 January 6]; Available from: <https://www.cancerresearchuk.org/health-professional/cancer-statistics/statistics-by-cancer-type/bowel-cancer>.

2. Cancer Research UK. *Breast Cancer Statistics*. [Internet] [cited 2022 January 6]; Available from: <https://www.cancerresearchuk.org/health-professional/cancer-statistics/statistics-by-cancer-type/breast-cancer>.

3. Cancer Research UK. *Oesophageal Cancer Statistics*. [Internet] [cited 2022 January 6]; Available from: <https://www.cancerresearchuk.org/health-professional/cancer-statistics/statistics-by-cancer-type/oesophageal-cancer>.

4. Cancer Research UK. *Liver Cancer Statistics*. [Internet] [cited 2022 January 6]; Available from: <https://www.cancerresearchuk.org/health-professional/cancer-statistics/statistics-by-cancer-type/liver-cancer>.

5. Cancer Research UK. *Head and Neck Cancer Statistics*. [Internet] [cited 2022 January 6]; Available from: <https://www.cancerresearchuk.org/health-professional/cancer-statistics/statistics-by-cancer-type/head-and-neck-cancers>.

6. Ratib, S., et al., *Diagnosis of liver cirrhosis in England, a cohort study, 1998-2009: A comparison with cancer.* American Journal of Gastroenterology, 2014. **109**(2): p. 190-198.

7. World Health Organization (WHO). *European Mortality Database (MDB)*. 2021 [cited 2022 January 17]; Available from: <https://gateway.euro.who.int/en/datasets/european-mortality-database/#:~:text=European%20mortality%20database%20allows%20age,Data%20reach%20back%20to%201980>.

8. British Heart Foundation (BHF). *Heart & Circulatory Disease Statistics 2020. [Internet]*. 2021 [cited 2022 January 6]; Available from: <https://www.bhf.org.uk/what-we-do/our-research/heart-statistics/heart-statistics-publications/cardiovascular-disease-statistics-2020>.

9. NHS Digital. *Health Survey for England, 2016*. 2017 [cited 2022 24th March]; Available from: <https://digital.nhs.uk/data-and-information/publications/statistical/health-survey-for-england/health-survey-for-england-2016>.

10. British Heart Foundation (BHF). *Heart & Circulatory Disease Statistics 2021*. 2022 [cited 2022 January 6]; Available from: <https://www.bhf.org.uk/what-we-do/our-research/heart-statistics/heart-statistics-publications/cardiovascular-disease-statistics-2021>.
