# Supplementary material for: Long-term health consequences and costs of changes in alcohol consumption in England during the COVID-19 pandemic
Source: PLoS One. 2025 Jan 16;20(1):e0314870. doi: 10.1371/journal.pone.0314870 (PMC11737736; doi:10.1371/journal.pone.0314870)
Supplement: S5 Table — (DOCX) [file pone.0314870.s006.docx]

S5 Table. Survival rates from disease data sources.

|  | Survival | | | | | |
| --- | --- | --- | --- | --- | --- | --- |
| Disease | **1-year (%)** | | **5-year (%)** | | **10-year (%)** | |
|  | **Male** | **Female** | **Male** | **Female** | **Male** | **Female** |
| Colorectal cancer  CRUK, 2021: Bowel cancer (C18-C20): 2013-2017 [11] | 79.3 | 77.1 | 58.2 | 58.6 | 51.9 | 53.8 |
| Breast cancer  CRUK, 2021: Breast cancer (C50): 2013-2017 [12] | NA | 95.8 | NA | 85.0 | NA | 75.9 |
| Oesophageal cancer  CRUK, 2021: Oesophageal cancer (C15): 2013-2017 [13] | 47.1 | 46.0 | 16.3 | 18.7 | 12.5 | 12.4 |
| Liver cancer  CRUK 2021: Liver cancer (C22): 2013-2017 [14] | 40.0 | 34.6 | 13.7 | 10.7 | NA | NA |
| Mouth cancer CRUK 2021: Oral Cavity Cancer (C03, C04, C05, C06): 2009-2013 [15] | 77.8 | 79.2 | 53.5 | 59.8 | 42.2 | 49.6 |
| Liver cirrhosis* |  |  |  |  |  |  |
| Throat cancer CRUK: Hypopharyngeal Cancer (C12, C13): 2009-2013 [15] | 60.4 | 60.7 | 27.2 | 30.2 | 17.9 | 23.3 |
| Stroke* |  |  |  |  |  |  |
| Hypertension (non-terminal) | _ | _ | _ | _ | _ | _ |

*Calculated from incidence, prevalence and mortality

## References

11. Cancer Research UK. *Bowel Cancer Survival Statistics*. [Internet] [cited 2022 January 9]; Available from: <https://www.cancerresearchuk.org/health-professional/cancer-statistics/statistics-by-cancer-type/bowel-cancer/survival>.

12. Cancer Research UK. *Breast Cancer Survival Statistics,*. [Internet] [cited 2022 January 9]; Available from: <https://www.cancerresearchuk.org/health-professional/cancer-statistics/statistics-by-cancer-type/breast-cancer/survival?_gl=1*1ydsd4b*_ga*ODU5Mzc0MDU4LjE2NDExNjI0OTY.*_ga_58736Z2GNN*MTY0MTc0MzE5Ni43LjEuMTY0MTc0MzkyNy41OQ..&_ga=2.178238241.525254709.1641743198-859374058.1641162496#heading-Zero>.

13. Cancer Research UK. *Oesophageal Cancer Survival Statistics*. [Internet] [cited 2022 January 9]; Available from: <https://www.cancerresearchuk.org/health-professional/cancer-statistics/statistics-by-cancer-type/oesophageal-cancer/survival#heading-Zero>.

14. Cancer Research UK. *Liver Cancer Survival Statistics*. [Internet] [cited 2022 January 9]; Available from: <https://www.cancerresearchuk.org/health-professional/cancer-statistics/statistics-by-cancer-type/liver-cancer/survival?_gl=1*1gahupr*_ga*ODU5Mzc0MDU4LjE2NDExNjI0OTY.*_ga_58736Z2GNN*MTY0MTc0MzE5Ni43LjEuMTY0MTc0NjIyNC42MA..&_ga=2.120109068.525254709.1641743198-859374058.1641162496>.

15. Cancer Research UK. *Head and Neck Cancer Survival Statistics*. [Internet] [cited 2022 January 9]; Available from: <https://www.cancerresearchuk.org/health-professional/cancer-statistics/statistics-by-cancer-type/breast-cancer/survival?_gl=1*1ydsd4b*_ga*ODU5Mzc0MDU4LjE2NDExNjI0OTY.*_ga_58736Z2GNN*MTY0MTc0MzE5Ni43LjEuMTY0MTc0MzkyNy41OQ..&_ga=2.178238241.525254709.1641743198-859374058.1641162496#heading-Zero>.
