# Supplementary material for: Long-term health consequences and costs of changes in alcohol consumption in England during the COVID-19 pandemic
Source: PLoS One. 2025 Jan 16;20(1):e0314870. doi: 10.1371/journal.pone.0314870 (PMC11737736; doi:10.1371/journal.pone.0314870)
Supplement: S6 Table — (DOCX) [file pone.0314870.s007.docx]

S6 Table. Relative risks in microsimulation data sources.

| Increase in risk relative to non-drinkers | | | |
| --- | --- | --- | --- |
| Disease | **Units per week*** | **Value (95% CI)** | |
|  |  | **Males** | **Females** |
| Colorectal Cancer Bagnardi et al. 2015 [16] | ≤10.9 | 1.05 (0.95-1.16) | 0.95 (0.89-1.01) |
|  | 11.0-43.8 | 1.21 (1.11-1.32) | 1.07 (0.99-1.16) |
|  | >43.8 | 1.53 (1.30-1.80) | 1.24 (0.68-2.25) |
| Breast Cancer Bagnardi et al. 2015 [16] | ≤10.9 | **-** | 1.04 (1.01-1.07) |
|  | 11.0-43.8 | **-** | 1.23 (1.19-1.28) |
|  | >43.8 | **-** | 1.61 (1.33-1.94) |
| Oesophageal Cancer Bagnardi et al. 2015 [16] | ≤10.9 | 1.39 (1.11-1.74) | 1.14 (0.87-1.49) |
|  | 11.0-43.8 | 2.25 (1.78-2.85) | 2.18 (1.42-3.35) |
|  | >43.8 | 4.69 (3.49-6.31) | 8.32 (2.95-23.45) |
| Liver Cancer Bagnardi et al. 2015 [16] | ≤10.9 | 1.05 (0.84-1.32) | 0.81 (0.59-1.12) |
|  | 11.0-43.8 | 1.08 (0.88-1.32) | 1.24 (0.88-1.75) |
|  | >43.8 | 1.59 (1.21-2.09) | 3.89 (1.6-9.48) |
| Mouth Cancer Bagnardi et al. 2015 [16] | ≤10.9 | 1.20 (1.06-1.25) | 1.00 (0.78-1.27) |
|  | 11.0-43.8 | 2.01 (1.69-2.40) | 1.67 (1.25-2.22) |
|  | >43.8 | 5.33 (4.28-6.63) | 5.70 (3.75-8.66) |
| Alcoholic Liver Disease Roerecke et al. 2019 [17] | 10-21 | 0.91 | 1.64 |
|  | >21-42 | 1.97 | 4.33 |
|  | >42-63 | 2.62 | 3.87 |
|  | >63-73 | 3.80 | 12.44 |
|  | >73 | 6.93 | 24.58 |
| Throat Cancer Bagnardi et al. 2015 [16] | ≤10.9 | 1.20 (1.06-1.25) | 1.00 (0.78-1.27) |
|  | 11.0-43.8 | 2.01 (1.69-2.40) | 1.67 (1.25-2.22) |
|  | >43.8 | 5.53 (4.28-6.63) | 5.70 (3.75-8.66) |
| Ischaemic Stroke Ronksley et al. 2011 [18] | ≤2.2 | 0.81 (0.74-0.89) | 0.81 (0.74-0.89) |
|  | 2.2-13.0 | 0.80 (0.74-0.87) | 0.80 (0.74-0.87) |
|  | 13.1-26.2 | 0.92 (0.82-1.04) | 0.92 (0.82-1.04) |
|  | 26.3-52.5 | 1.15 (0.98-1.35) | 1.15 (0.98-1.35) |
|  | >52.5 | 1.62 (1.32-1.98) | 1.62 (1.32-1.98) |
| Hypertension Taylor et al. 2009 [19] | >0-4 | 0.762499988 | 0.730700016 |
|  | >4-8 | 0.828499973 | 0.832780004 |
|  | >8-10 | 0.896099985 | 0.934220016 |
|  | >10-14 | 0.930499971 | 0.984700024 |
|  | >14-18 | 1.000499964 | 1.085180044 |
|  | >18-21 | 1.072100043 | 1.185019970 |
|  | >21-30 | 1.126850009 | 1.259480000 |
|  | >30-35 | 1.296499968 | 1.480700016 |
|  | >35-40 | 1.394250035 | 1.602200031 |
|  | >40-45 | 1.494500041 | 1.722700000 |
|  | >45-50 | 1.597249985 | 1.842200041 |
|  | >50-55 | 1.702499986 | 1.960700035 |
|  | >55-60 | 1.810250044 | 2.078200102 |
|  | >60-65 | 1.920500040 | 2.194700003 |
|  | >65-70 | 2.033250093 | 2.310199976 |
|  | >70-75 | 2.148499966 | 2.424700022 |
|  | >75-80 | 2.266249895 | 2.538199902 |
|  | >80-85 | 2.386499882 | 2.650700092 |
|  | >85-90 | 2.509249926 | 2.762200117 |
|  | >90-95 | 2.634500027 | 2.872699976 |
|  | >95-100 | 2.762249947 | 2.982199907 |
|  | >100-110 | 2.892499924 | 3.090699911 |
|  | >110-120 | 3.160500050 | 3.304699898 |
|  | >120-130 | 3.438499928 | 3.514699936 |
|  | >130-140 | 3.726500034 | 3.720700026 |
|  | >140-150 | 4.024499893 | 3.922699928 |
|  | >150 | 4.332499981 | 4.120699883 |

References

16. Bagnardi, V., et al., *Alcohol consumption and site-specific cancer risk: a comprehensive dose-response meta-analysis.* Br J Cancer, 2015. **112**(3): p. 580-93.

17. Roerecke, M., et al., *Alcohol Consumption and Risk of Liver Cirrhosis: A Systematic Review and Meta-Analysis.* Am J Gastroenterol, 2019. **114**(10): p. 1574-1586.

18. Ronksley, P.E., et al., *Association of alcohol consumption with selected cardiovascular disease outcomes: a systematic review and meta-analysis.* Bmj, 2011. **342**: p. d671.

19. Taylor, B., et al., *Alcohol and hypertension: gender differences in dose-response relationships determined through systematic review and meta-analysis.* Addiction, 2009. **104**(12): p. 1981-90.
