# Supplementary material for: Long-term health consequences and costs of changes in alcohol consumption in England during the COVID-19 pandemic
Source: PLoS One. 2025 Jan 16;20(1):e0314870. doi: 10.1371/journal.pone.0314870 (PMC11737736; doi:10.1371/journal.pone.0314870)
Supplement: S8 Table — (DOCX) [file pone.0314870.s009.docx]

S8 Table. Cost of breast cancer data sources.

|  | Direct health cost (Laudicella et al. 2016 [20]) |
| --- | --- |
| Cost cited | Costs over 9 year period from diagnosis  £26,304.00 per patient 18-64 years (for 2010)  £25,966.00 per patient 65+ years (for 2010) |
| Definition | - Costs in the year of diagnosis - Includes: surgery cost, inpatient and outpatient hospital cost in the first 12 months after diagnosis - Based on costs of hospital activity fixed in 2010 |
| Cost used in the microsimulation (2021) | £3644.80 |
| Cost calculation | The average for both age groups, divided by 9 years, inflated to 2021 |

Reference

20. Laudicella, M., et al., *Cost of care for cancer patients in England: evidence from population-based patient-level data.* British Journal of Cancer, 2016. **114**(11): p. 1286-1292.
