# Supplementary material for: Long-term health consequences and costs of changes in alcohol consumption in England during the COVID-19 pandemic
Source: PLoS One. 2025 Jan 16;20(1):e0314870. doi: 10.1371/journal.pone.0314870 (PMC11737736; doi:10.1371/journal.pone.0314870)
Supplement: S10 Table — (DOCX) [file pone.0314870.s011.docx]

S10 Table. Cost of liver cancer data sources.

|  | Direct health cost for HCC* (McEwan et al. 2017 [22]) |
| --- | --- |
| Cost cited | £10,451.58 (SE** £2456.09) inflated to 2013 values |
| Definition | - Mean cost for HCC in 2012-2013 |
| Cost used in the microsimulation (2021) | £12,016.24 |
| Cost calculation | Inflated to 2021 |

* Hepatocellular carcinoma, ** Standard error

Reference

22. McEwan, P., et al., *Estimating the cost-effectiveness of daclatasvir + sofosbuvir versus sofosbuvir + ribavirin for patients with genotype 3 hepatitis C virus.* Cost Eff Resour Alloc, 2017. **15**: p. 15.
