# Supplementary material for: Long-term health consequences and costs of changes in alcohol consumption in England during the COVID-19 pandemic
Source: PLoS One. 2025 Jan 16;20(1):e0314870. doi: 10.1371/journal.pone.0314870 (PMC11737736; doi:10.1371/journal.pone.0314870)
Supplement: S11 Table — (DOCX) [file pone.0314870.s012.docx]

S11 Table. Cost of mouth cancer data sources.

|  | Direct health cost (Speight et al. 2006 [23]) |
| --- | --- |
| Cost cited | £1,869.00 precancer  £4,914.00 stage I  £8,535.00 stage II  £11,883.00 stage III  £13,513.00 stage IV (all values for year 2002-2003) |
| Definition | The total cost per patient over the 3-year study period (diagnosed in years 1998-2000) |
| Cost used in the microsimulation (2021) | £7011.17 |
| Cost calculation | Average taken assuming equal distribution of stages, inflated to 2021 |

Reference

23. Speight, P.M., et al., *The cost-effectiveness of screening for oral cancer in primary care.* Health Technol Assess, 2006. **10**(14): p. 1-144, iii-iv.
