# Supplementary material for: Long-term health consequences and costs of changes in alcohol consumption in England during the COVID-19 pandemic
Source: PLoS One. 2025 Jan 16;20(1):e0314870. doi: 10.1371/journal.pone.0314870 (PMC11737736; doi:10.1371/journal.pone.0314870)
Supplement: S12 Table — (DOCX) [file pone.0314870.s013.docx]

S12 Table. Cost of liver cirrhosis data sources.

|  | Direct health cost (Tanajewski et al. 2017 [24]) |
| --- | --- |
| Cost cited | £1218.65 (cost inflated to 2013-2014) |
| Definition | Annual cost per patient for SLD* state, including cost of test, primary care, secondary care, other (dietitian), and medication |
| Cost used in the microsimulation (2021) | £1377.52 |
| Cost calculation | Inflated to 2021 |

* Significant liver disease (fibrosis stage 2 or 3)

Reference

24. Tanajewski, L., et al., *Economic evaluation of a community-based diagnostic pathway to stratify adults for non-alcoholic fatty liver disease: a Markov model informed by a feasibility study.* BMJ Open, 2017. **7**(6): p. e015659.
