# Supplementary material for: Long-term health consequences and costs of changes in alcohol consumption in England during the COVID-19 pandemic
Source: PLoS One. 2025 Jan 16;20(1):e0314870. doi: 10.1371/journal.pone.0314870 (PMC11737736; doi:10.1371/journal.pone.0314870)
Supplement: S13 Table — (DOCX) [file pone.0314870.s014.docx]

S13 Table. Cost of throat cancer data sources.

|  | Direct health cost (Agus et al. 2013 [21]) |
| --- | --- |
| Cost cited | £7,847.00 (for 2005) |
| Definition | Average cost of hospital care per patient in 12 months from presentation of oesophageal cancer |
| Cost used in the microsimulation (2021) | £10,654.29 |
| Cost calculation | Inflated to 2021 |

Reference

21. Agus, A.M., et al., *Description and predictors of hospital costs of oesophageal cancer during the first year following diagnosis in Northern Ireland.* Eur J Cancer Care (Engl), 2013. **22**(4): p. 450-458.
