# Supplementary material for: Long-term health consequences and costs of changes in alcohol consumption in England during the COVID-19 pandemic
Source: PLoS One. 2025 Jan 16;20(1):e0314870. doi: 10.1371/journal.pone.0314870 (PMC11737736; doi:10.1371/journal.pone.0314870)
Supplement: S14 Table — (DOCX) [file pone.0314870.s015.docx]

S14 Table. Cost of stroke data sources.

|  | Direct health cost (Ward et al. [25]) |
| --- | --- |
| Cost cited | £6,120.00 (cost in 1^st^ year)  £2,815.00 (cost in 2^nd^ year) (both values for 2007) |
| Definition | The annual cost for a treatment of a single stroke patient based on the weighted distribution of mild, moderate, and severe stroke costs, excluding event cost |
| Cost used in the microsimulation (2021) | £3,520.99 |
| Cost for second year | Cost for second year inflated to 2021 |

Reference

25. Ward, S., et al., *A systematic review and economic evaluation of statins for the prevention of coronary events.* Health Technology Assessment, 2007. **11**(14).
