# Supplementary material for: Long-term health consequences and costs of changes in alcohol consumption in England during the COVID-19 pandemic
Source: PLoS One. 2025 Jan 16;20(1):e0314870. doi: 10.1371/journal.pone.0314870 (PMC11737736; doi:10.1371/journal.pone.0314870)
Supplement: S15 Table — (DOCX) [file pone.0314870.s016.docx]

S15 Table. Cost of hypertension data sources.

|  | Direct health cost [26] and [27] |
| --- | --- |
| Cost cited | See definition |
| Cost used in the microsimulation (2021) | £153.12 |
| Cost calculation | - The average annual cost of monitoring hypertension via consultations and clinical tests from the second year onwards (£75.00, uninflated), in addition to the average annual cost of hypertension treatment (£57.20, uninflated) based on the use of the most common generic drug in each glass, accounting for the proportion of people on different drug classes and those taking combination therapies. Cost inflated to 2021. |

References

26. Constanti, M., et al., *Cost-Effectiveness of Initiating Pharmacological Treatment in Stage One Hypertension Based on 10-Year Cardiovascular Disease Risk: A Markov Modeling Study.* Hypertension, 2021. **77**(2): p. 682-691.

27. Lovibond, K., et al., *Cost-effectiveness of options for the diagnosis of high blood pressure in primary care: a modelling study.* Lancet, 2011. **378**(9798): p. 1219-30.
